# Lack of Association between the *TSPAN18* Gene and Schizophrenia Based on New Data from Han Chinese and a Meta-Analysis

**DOI:** 10.3390/ijms160611864

**Published:** 2015-05-26

**Authors:** Bao Zhang, Da-Xu Li, Ning Lu, Qian-Rui Fan, Wen-Hao Li, Zu-Fei Feng

**Affiliations:** 1 College of Forensic Science, Health Science Center, Xi’an Jiaotong University, Xi’an 710061, China; E-Mails: zhangbao_814@mail.xjtu.edu.cn (B.Z.); fanqianrui@stu.xjtu.edu.cn (Q.-R.F.); liwenhao@stu.xjtu.edu.cn (W.-H.L.); 2 Department of Stomatology, the First Affiliated Hospital, Xi’an Jiaotong University, Xi’an 710061, China; E-Mail: sheraer@gmail.com; 3 Department of Food and Biological Engineering, Henan Industry & Trade Vocational College, Zhengzhou 451191, China; E-Mail: forensics@mail.xjtu.edu.cn

**Keywords:** *TSPAN18* gene, single nucleotide polymorphisms, schizophrenia, association, meta

## Abstract

Tetraspanin-18 (*TSPAN18*) potentially plays a role in the calcium signaling that is associated with dopamine-induced cortical neuron apoptosis and is considered to be an important mechanism in the pathogenesis of schizophrenia (SCZ). Furthermore, a genome-wide association study (GWAS) identified *TSPAN18* as a possible susceptibility gene for SCZ. To validate these findings and reveal the effects of different inheritance models, seven single nucleotide polymorphisms (SNPs) of the *TSPAN18* gene were analyzed in 443 patients with SCZ and 628 controls of Han Chinese descent via the SNPscan method. Single SNP, genotype, and association analyses with different models (*i.e.*, additive, dominant, and recessive models) were performed, and the published datasets (2062 cases and 2053 controls) were combined with our results to determine the inheritance effects of the SNPs on SCZ. We observed genotypes and allele distributions of *TSPAN18* gene did not show any significant associations in the Han Chinese population based on our experimental and meta-analytical results. Our findings indicate that the *TSPAN18* gene is unlikely to be a major susceptibility gene for schizophrenia in Han Chinese.

## 1. Introduction

Schizophrenia (SCZ) is a common, severe, and debilitating mental disorder that is influenced by both genetic and environmental factors [1]. It has been reported that approximately 1% of the general population worldwide is affected by SCZ [2,3], and the morbidity among males is greater than among females [4]. Although heritability has been proven to play an important role in the development of SCZ [5], the exact etiology and genetic mechanisms of SCZ remain unclear.

Previously, many candidate genes, including *NRG1* (encoding neuregulin 1), *AUTS2* (autism susceptibility candidate 2), *TSPAN8* (Tetraspanin-8), *ZNF804A* (zinc-finger protein 804A), among others, have been identified that might confer risk for schizophrenia [6,7,8,9]. In recent years, a genome-wide association study (GWAS) of schizophrenia have identified several susceptibility loci, including ZKSCAN4 (zinc finger with KRAB and SCAN domains 4), NKAPL (NFκB activating protein-like), MIR137 (microRNA 137), WDR1 (WD repeat-containing protein 1), the ITIH3 (inter-alpha-trypsin inhibitor heavy chain 3)–ITIH4 (inter-alpha-trypsin inhibitor heavy chain family, member 4) region and genes within the extended major histocompatibility complex (MHC) region [10,11,12]. Among these genes, *TSPAN18* gene with three single nucleotide polymorphisms (SNPs) (rs11038167, rs11038172, and rs835784) was reported to increase susceptibility to SCZ among Han Chinese [6]. Yuan *et al.* also found a significant association between rs835784 and SCZ [7]; however, others have failed to confirm this association [8]. Foremost, the genetic model based on previous studies is scarce. Given these controversial results regarding the association of the *TSPAN18* gene with SCZ, the contribution of this gene to the etiology of the disorder requires further clarification.

Therefore, to examine the association of *TSPAN18* with SCZ further, and to reveal the inheritance models for the association results, we conducted a case-control study involving cases from the Northwest Han Chinese population. Additionally, a meta-analysis of the SNPs of the examiner gene was performed across the published studies and the current research.

## 2. Results

### 2.1. Case-Control Study

**Figure 1:**
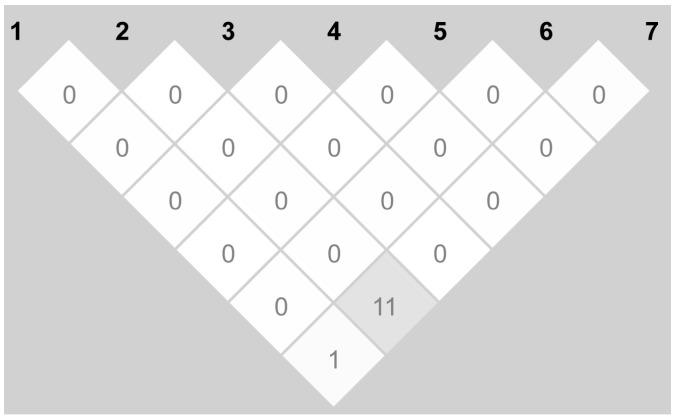
Linkage disequilibrium (LD) plot of 7 single nucleotide polymorphisms (SNPs) of the *TSPAN18* (Tetraspanin-8) gene. The values in the squares indicate the pair-wise calculations of *R*^2^. The white squares with “0” indicate *R*^2^ = 0 (*i.e.*, no LD between a pair of SNPs).

Seven SNPs in the *TSPAN18* gene were genotyped in 443 SCZ cases and 628 controls. No significant deviations from Hardy–Weinberg equilibrium (HWE) were found in either group. The frequencies of the alleles and genotypes of the seven SNPs are shown in Table 1. When all of the samples were considered, the allele frequencies and genotype association analysis for the patients with SCZ did not significantly differ from those of the control group. After the linkage disequilibrium (LD) blocks were created, we found that seven SNPs exhibited very low *R*^2^ values (Figure 1), indicating that the calculations of the haplotype frequencies were not required.

**Table 1:** Allele and genotype frequencies in the single SNP association analyses.

Makers		Allele Freq (%)		*p*-Value	OR ^a^ (95% CI)	Genotype (N)			HWE *p*-Value	Model	OR ^b^ (95% CI)	*p*-Value
SNP	ID											
SNP1	rs11038167	A	C			AA	AC	CC				
SCZ		43.5	56.5	0.935	0.993 (0.835–1.181)	79	227	137	0.238	Add	0.993 (0.838–1.177)	0.936
CTR		43.6	56.4			134	280	214		Dom	1.155 (0.890–1.498)	0.297
										Rec	0.800 (0.587–1.090)	0.157
SNP2	rs11038172	A	G			AA	AG	GG				
SCZ		47.9	52.1	0.166	1.130 (0.951–1.342)	114	197	132	0.954	Add	1.127 (0.950–1.337)	0.171
CTR		44.8	55.2			120	323	185		Dom	0.984 (0.754–1.284)	0.905
										Rec	1.450 (1.083–1.941)	0.012
SNP3	rs835990	G	A			GG	AG	AA				
SCZ		25.2	74.8	0.796	1.027 (0.841–1.254)	31	158	248	0.461	Add	1.026 (0.843–1.249)	0.799
CTR		24.7	75.3			41	224	355		Dom	1.021 (0.797–1.307)	0.870
										Rec	1.078 (0.665–1.749)	0.760
SNP4	rs704671	A	C			AA	AC	CC				
SCZ		37.4	62.6	0.269	1.106 (0.925–1.323)	58	215	170	0.324	Add	1.104 (0.925–1.318)	0.274
CTR		35.0	65.0			86	268	274		Dom	1.243 (0.970–1.593)	0.086
										Rec	0.949 (0.664–1.358)	0.776
SNP5	rs73456450	G	A			GG	AG	AA				
SCZ		2.25	97.75	0.667	0.883 (0.502–1.555)	0	20	423	0.551	Add	0.889 (0.512–1.543)	0.676
CTR		2.25	97.75			2	28	598		Dom	0.943 (0.528–1.682)	0.841
										Rec		0.999
SNP6	rs836001	G	C			GG	GC	CC				
SCZ		25.3	75.7	0.739	0.967 (0.794–1.178)	39	146	258	0.065	Add	0.969 (0.799–1.174)	0.746
CTR		25.9	75.1			43	237	343		Dom	0.878 (0.687–1.124)	0.302
										Rec	1.302 (0.829–2.045)	0.252
SNP7	rs836002	G	C			GG	GC	CC				
SCZ		24.2	75.8	0.716	0.964 (0.789–1.177)	30	154	259	0.621	Add	0.964 (0.970–1.176)	0.718
CTR		24.8	75.2			37	238	353		Dom	0.912 (0.713–1.166)	0.463
										Rec	1.160 (0.705–1.908)	0.558

Allele freq: allele frequency; SCZ: schizophrenia; CTR: control; CI: confidence interval; OR: odds ratio; ^a^: OR refers to risk allele odds ratio in cases and controls; ^b^: OR refers to risk genotype (Add: additive model; Dom: dominant model; Rec: recessive model) odds ratio in cases and controls.

### 2.2. Meta-Analysis

**Figure 2:**
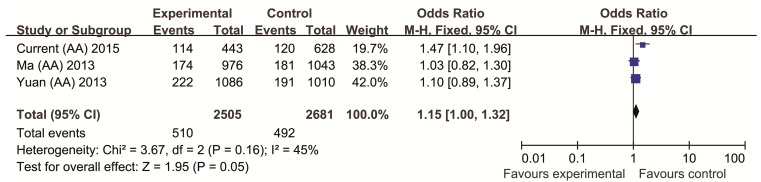
Forest plot of the *TSPAN18* rs11038172 polymorphism and Schizophrenia (SCZ) risk in the overall population.

For the meta-analysis, we combined and analyzed the data from two previous studies with those from the current study for totals of 2505 cases and 2681 controls [7,8]. A statistical summary of the meta-analysis of rs11038172 is shown in Figure 2.

Heterogeneity was found to be present between the three studies (*x*^2^ = 3.67, *p* = 0.16, heterogeneity = 0.45); therefore, the fixed effects model was employed. The pooled OR and 95% CI values were calculated for the rs11038172 polymorphism of the *TSPAN18* gene. The association between the rs11038172 polymorphism and SCZ risk in the recessive model showed *p*-value with 0.05 (pooled OR = 1.15, 95% CI, 1.00–1.32, *p* = 0.05).

## 3. Discussion

Tetraspanins are a highly conserved superfamily of four-transmembrane proteins that regulate diverse processes, such as cell signaling and adhesion, intracellular trafficking, viral infection and cancer [9]. The over-expressions of some tetraspanins have been demonstrated to be correlated with tumor progression [10,11]. Notably, studies found some tetraspanins to be acting in the nervous system during development and in the adult [12,13]. A previous study revealed that the *TSPAN18* potentially exerted a role in calcium signaling [14]. Moreover, the calcium-signaling pathway that was associated with dopamine-induced cortical neuron apoptosis had been considered to be an important mechanism in SCZ pathogenesis [15]; Furthermore, *TSPAN18* gene was reported to increase the susceptibility to SCZ among Han Chinese [6]. Yuan *et al.* also found a significant association between rs835784 and SCZ [7]; however, others have failed to confirm this association [8].

In our study, we observed genotypes and allele distributions of rs11038167 and rs11038172 SNPs in *TSPAN18* gene did not show any significant associations in Chinese population, which was consistent with previous studies [7,8]. In addition, we performed the present meta-analysis on the combined data from two previous studies and those of the current study, which comprised a total of 2505 cases and 2681 controls. Our findings revealed that the *TSPAN18* rs11038167 did not show any significant result (data not shown), and rs11038172 polymorphism showed *p*-value with 0.05 under recessive model (Figure 2). However, the recessive model based on our sample sizes could easily cause statistical type I error. Furthermore, the significant level was too weak in our meta-analysis result, implying there was no significant association between *TSPAN18* polymorphism and schizophrenia susceptibility in Chinese Han population.

Although a previous study revealed that *TSPAN18* was associated with SCZ, our experimental results and meta-analytical results do not support this finding. These inconsistent findings might have resulted from several factors. The frequencies of the “A” allele in rs11038167 in the controls from our studies, the studies of Yue and Yuan, and the European data from the NCBI were 0.448, 0.418, 0.430, and 0.991, respectively [6,7]. These differences in *TSPAN18* indicated that subjects from different regions might exhibit genetic heterogeneity related to SCZ, and this supposition has also been supported by a meta-analysis [16]. Furthermore, one study reported that different genotyping methodologies might affect observed allele frequencies and thereby cause heterogeneity [17]. Additionally, the possibility of sampling error due to differences in the clinical diagnoses of SCZ cannot be entirely being excluded.

Our study also has several limitations, particularly the relatively small sample size of our experimental study, which limits the interpretation of the results. Furthermore, the population information in this study was acquired via self-reported questionnaires; therefore, we cannot fully exclude the possibility that inaccurate information was responsible for the observed association. Additionally, this study was performed at a single center (the three populations of the meta-analysis were all from China), which might potentially limit the generalizability of the findings.

In summary, the results of our experimental and meta-analyses are unlikely to explain genetic effects on the risk of schizophrenia. However, due to genetic heterogeneity, further studies utilizing greater numbers of subjects from different ethnic groups and should be performed to clarify this association further.

## 4. Experimental Section

### 4.1. Subjects

Our samples were collected from the First Affiliated Hospital of Xi’an Jiaotong University. All patients were assigned by a standard procedure, and the diagnoses of SCZ were confirmed by two experienced psychiatrists using the Statistical Manual of Mental Disorders, Fourth Edition (DSM-IV). After undergoing a health examination, the controls were confirmed to be free of mental illness and matched with the patients in terms of sex, age, origin and educational level. Together, 443 patients (224 female and 219 male, mean age: 36.1 ± 10.2 years) and 628 unrelated controls (358 female and 270 male, mean age: 35.7 ± 9.7 years) of Northwestern Han Chinese descent were selected. All participants were volunteers, longstanding residents of the Shanxi province and provided written informed consent prior to inclusion. The study protocol was approved by the institutional review board of the Xi’an Jiaotong University Health Science Center with project identification code (2011-054).

### 4.2. Single Nucleotide Polymorphism (SNP) Selection and Genotyping

In the current study, we conducted preliminary analysis using the HapMap data to select the transcription unit (including the 5'UTR, exons, and 3'UTR regions) of the *TSPAN18* gene regions tagSNPs. The selected tagSNPs met the following criteria [18]. First, we examined tagSNPs in Haploview (v4.2) using the CHB (Chinese Han Beijing) population and a minor allele frequency cut-off (MAF) > 5%. Second, a MAF > 20% with pair-wise tagging and *R*^2^ > 0.8 [19] were used as cut-off for the selection of tagSNPs. Ultimately, seven SNPs (rs11038167, intron 2; rs11038172, intron 2; rs835990, intron 8; rs704671, exon10; rs73456450, exon10; rs836001, exon10; and rs836002, exon10) were selected for genotyping and analyses.

Human genomic DNA was extracted according to the manufacturer’s recommendations (Omiga, Bio-tek, Norcross, GA, USA). All SNPs were genotyped with the SNPscan technique using SNPscan™ kit (Genesky Biotechnologies Inc., Shanghai, China) to design and determine the genotypes. This is a high-throughput and cost-saving SNP genotyping method based on double ligation and multiplex fluorescence PCR [20,21], and its high accuracy has been validated by many studies [22,23]. Five percent of the high DNA quality samples were randomly subjected to repeated analyses to guarantee the genotyping qualities. The average genotype call rate for all markers was 96%.

### 4.3. Statistical Analyses

The statistic power of our sample size was calculated with the G*Power program (Franz Faul, University Kiel, Kiel, Germany), according to Cohen’s method [24]. The sample size indicated a > 90% power to detect significant (a < 0.05) associations of alleles, genotypes and haplotypes at an effect size of 0.1 (which corresponds to a “weak” gene effect).

The genotype, allele and haplotype frequency differences between the cases and controls were calculated with chi-square test, which was also used to estimate whether the SNP genotypes deviated from the expected Hardy–Weinberg equilibrium (HWE) values. Logistic regression analyses were selected to identify the SCZ-associated SNPs according to the odds ratios (OR), 95% confidence intervals (CIs), and corresponding *p*-values. Moreover, the analyses of single SNPs were performed using the following multiple inheritance models: the additive, dominant model (the minor allele homozygotes plus the heterozygotes *vs.* the major allele homozygotes), and a recessive model (the minor allele homozygotes *vs.* the heterozygotes plus the major allele homozygotes).

Partitioning of the linkage disequilibrium (LD) blocks was performed with Haploview 4.2 [25]. All statistical analyses were performed with PLINK (version 1.07) [26]. Differences were considered significant when the *p* value was <0.007 after Bonferroni correction (0.05/7).

### 4.4. Meta-Analysis

The studies included in the meta-analysis were identified using the MEDLINE, Web of Science, and Pubmed databases using the following keywords “TSPAN18” and “Schizophrenia”. Data from two previously published papers were combined with ours’ from the present study [7,8]. We used Cochrane Collaboration RevMan 5 to conduct case-control meta-analyses of independent samples under dominant and recessive models. Chi-squared tests based on *Q* tests and *I*^2^ were used to assess the heterogeneities between the individual studies [27,28,29]. When *p* < 0.05 (Q test) or *I*^2^ > 50%, the random effects model (the DerSimonian & Laird method) was employed [30]. Otherwise, the fixed effects model (the Mantel–Haenszel method) was used [31]. All *p*-values were two-tailed, and the significance level was 0.05.
